# O10: Real-time magnetic resonance cine imaging with compressed sensing and iterative reconstruction for ventricular measures: comparison with gold-standard segmented steady-state free precession

**DOI:** 10.1186/1532-429X-16-S1-O10

**Published:** 2014-01-16

**Authors:** Gabriel Camargo, Leticia R Sabioni, Thomas Doring, Ralph Strecker, Maria Eduarda Derenne, Vania M Naue, Tamara Rothstein, Michaela Schmidt, Michael O Zenge, Mariappan S Nadar, Ronaldo S Lima, Ilan Gottlieb

**Affiliations:** 1 CDPI, Rio de Janeiro, Brazil; 2 Siemens LTDA, São Paulo, Brazil; 3 Healthcare Sector, Siemens AG, Erlangen, Germany; 4 Siemens Corporate Technology, Princeton, New Jersey, USA

## Background

Segmented cine imaging with a steady-state free precession sequence (CINE-SSFP) is currently the gold standard technique for measuring ventricular volumes and mass. It requires multiple breath-holds to cover the entire ventricles, thus being prone to misalignment of consecutive slices, time consuming and dependent on breath-hold (BH) capability. Real-time cine avoids those limitations, however poor spatial and temporal resolution of conventional sequences have prevented its routine application. We sought to examine if a newly developed real-time sequence featuring compressed sensing and iterative reconstruction (CINE-RT), which is an investigational prototype, would yield similar results when compared with conventional CINE-SSFP in a group of healthy volunteers.

## Methods

Stacks of short-axis cines were acquired covering both ventricles in a 1.5T system (MAGNETOM Aera, Siemens, Germany), using gold standard CINE-SSFP and CINE-RT. Acquisition parameters for CINE-SSFP were: voxel size 1,6 × 1,6 × 7,0 mm, GRAPPA acceleration factor of 2, temporal resolution of 39 ms, retrospective gating, with an average of 8 heart beats per slice and 1 slice/BH. For CINE-RT: voxel size 1,6 × 1,6 × 7,0 mm, compressed sensing acceleration factor of 10, temporal resolution of 41 ms, prospective gating, real-time acquisition of 1 heart-beat/slice and all slices in one BH. CINE-RT images were repeated three times using different degrees of iterations (40, 60 and 80) to explore the accuracy vs. reconstruction time relationship. Left and right ventricle contours were blindly drawn by an experienced observer at end diastole and systole to derive volumes and LV mass. Comparisons were made using Pearson's correlation coefficients and Bland-Altman plots.

## Results

**Figure 1:**
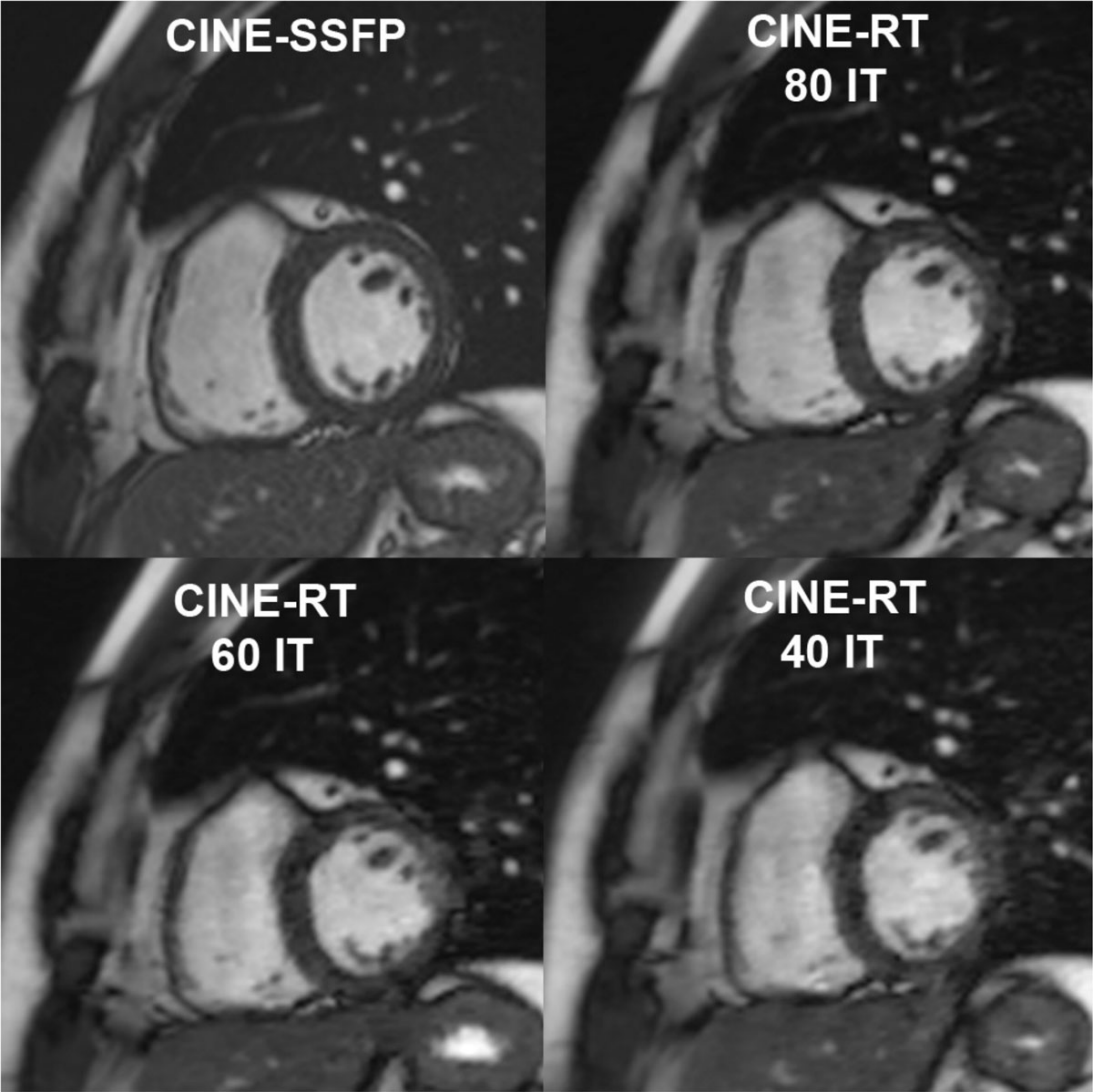
**IT: reconstruction iterations, other abbreviations as in text**.

Eight healthy volunteers (4 male; 35.2 ± 4.5 years) were examined in the same day. All subjects were in sinus rhythm and all images were considered to have diagnostic quality (Figure 1). CINE-RT derived volumes and mass correlated with gold standard CINE-SSFP, with small biases. Higher amounts of reconstruction iterations from 40 to 80 were related to decreased measurement biases. Table 1 summarizes all results and comparisons.

**Table 1:** 

	LV EDV			LV ESV			LV Mass			RV EDV			RV ESV		
	
	ml ± SD	r	bias ± SD	ml ± SD	r	bias ± SD	g ± SD	r	bias ± SD	ml ± SD	r	bias ± SD	ml ± SD	r	bias ± SD
CINE-SSFP	134.5 ± 20.2	-	-	47.3 ± 13.0	-	-	82.5 ± 17.7	-	-	127.0 ± 29.0	-	-	58.2 ± 19.3	-	-
CINE-RT 80 IT	122.0 ± 17.6	0.96	12.5 ± 6.0	47.5 ± 17.4	0.95	-0.2 ± 6.4	79.0 ± 18.9	0.87	3.5 ± 9.8	120.8 ± 24.5	0.97	6.2 ± 8.3	66.2 ± 16.0	0.94	-8.0 ± 7.1
CINE-RT 60 IT	121.2 ± 19.7	0.94	13.3 ± 6.8	47.3 ± 18.4	0.95	0.0 ± 7.4	76.5 ± 17.6	0.87	6.0 ± 8.9	119.0 ± 23.4	0.88	8.0 ± 5.8	65.8 ± 20.0	0.95	-7.7 ± 6.5
CINE-RT 40 IT	120.5 ± 16.8	0.83	14.0 ± 11.2	47.5 ± 16.1	0.98	-0.2 ± 4.2	73.7 ± 18.9	0.88	8.8 ± 8.9	115.3 ± 18.0	0.90	11.7 ± 15.2	68.0 ± 18.5	0.90	-9.8 ± 8.7

LV: lef ventricle, RV: right ventricle, EDV: end-diastolic volume, ESV: end-systolic volume, SD: standard deviation, IT: iterations, r: correlation coefficient (vs. CINE-SSFP), bias: mean bias (vs. CINE-SSFP).

## Conclusions

In our small pilot study of normal volunteers, CINE-RT with compressed sensing and iterative reconstruction with 1 heart beat per slice achieved spatial and temporal resolutions equivalent to CINE-SSFP, yielding correlated measures of ventricular volumes and mass. Higher number of iterations seem to provide more accurate results.

## Funding

Internal.

